# The therapeutic potential of Apigenin in amyotrophic lateral sclerosis through ALDH1A2/Nrf2/ARE signaling

**DOI:** 10.1186/s10020-024-00977-7

**Published:** 2024-11-09

**Authors:** Huiting Liang, Xinhui Zhou, Jie Zhang, Wenyuan Xu, Yi Liu, Xinxin Wang, Yushu Hu, Renshi Xu, Xiaobing Li

**Affiliations:** 1 Department of Neurology, First Affiliated Hospital of Nanchang University, Nanchang, 330006 China; 2 Institute of Neurology, Jiangxi Academy of Clinical Medical Science, The First Affiliated Hospital, Jiangxi Medical College, Nanchang University, Nanchang, 330006 China; 3 Rare Disease Center, The First Affiliated Hospital, Jiangxi Medical College, Nanchang University, Nanchang, 330006 China; 4 Key Laboratory of Rare Neurological Diseases of Jiangxi Provincial Health Commission, Jiangxi Medical College, Nanchang University, Nanchang, 330006 China; 5 Department of Neurosurgery, First Affiliated Hospital of Nanchang University, Nanchang, 330006 China; 6 Department of Neurology, Jiangxi Provincial People’s Hospital, Nanchang, 330006 China

**Keywords:** SOD1*G93A, Amyotrophic lateral sclerosis, Apigenin, ALDH1A2, Oxidative stress

## Abstract

**Background:**

Amyotrophic lateral sclerosis (ALS) is a progressive neurodegenerative disease characterized by motor neuron loss leading to muscle weakness and atrophy. Apigenin (APG), known for its antioxidant properties, holds potential as a therapeutic compound in ALS.

**Methods:**

We used the Tg(SOD1*G93A)1Gur/J transgenic mouse model of ALS to investigate the therapeutic effects of APG. Key measured included motor function via the ALSTDI score, molecular markers of oxidative stress (OS) and apoptosis in spinal cord tissues. Techniques used included pathological, Western blotting, flow cytometry, and qRT-PCR to assess the effect of ALDH1A2.

**Results:**

APG treatment attenuated weight loss and improved motor function scores in ALS mice compared to untreated ALS models. Molecular analyses revealed a significant upregulation of ALDH1A2 in APG-treated groups, along with a reduction in markers of OS and apoptosis. In vitro studies in NSC34 cells further confirmed the protective effects of APG against SOD1*G93A mutation-induced cytotoxicity. In addition, suppression of ALDH1A2 by shRNA exacerbated disease markers that were ameliorated by APG treatment.

**Conclusions:**

Our results suggest that APG attenuates the progression of ALS pathology by regulating OS and apoptosis through ALDH1A2. These results support further investigation of APG as a potential therapeutic agent for the treatment of ALS.

**Graphical Abstract:**

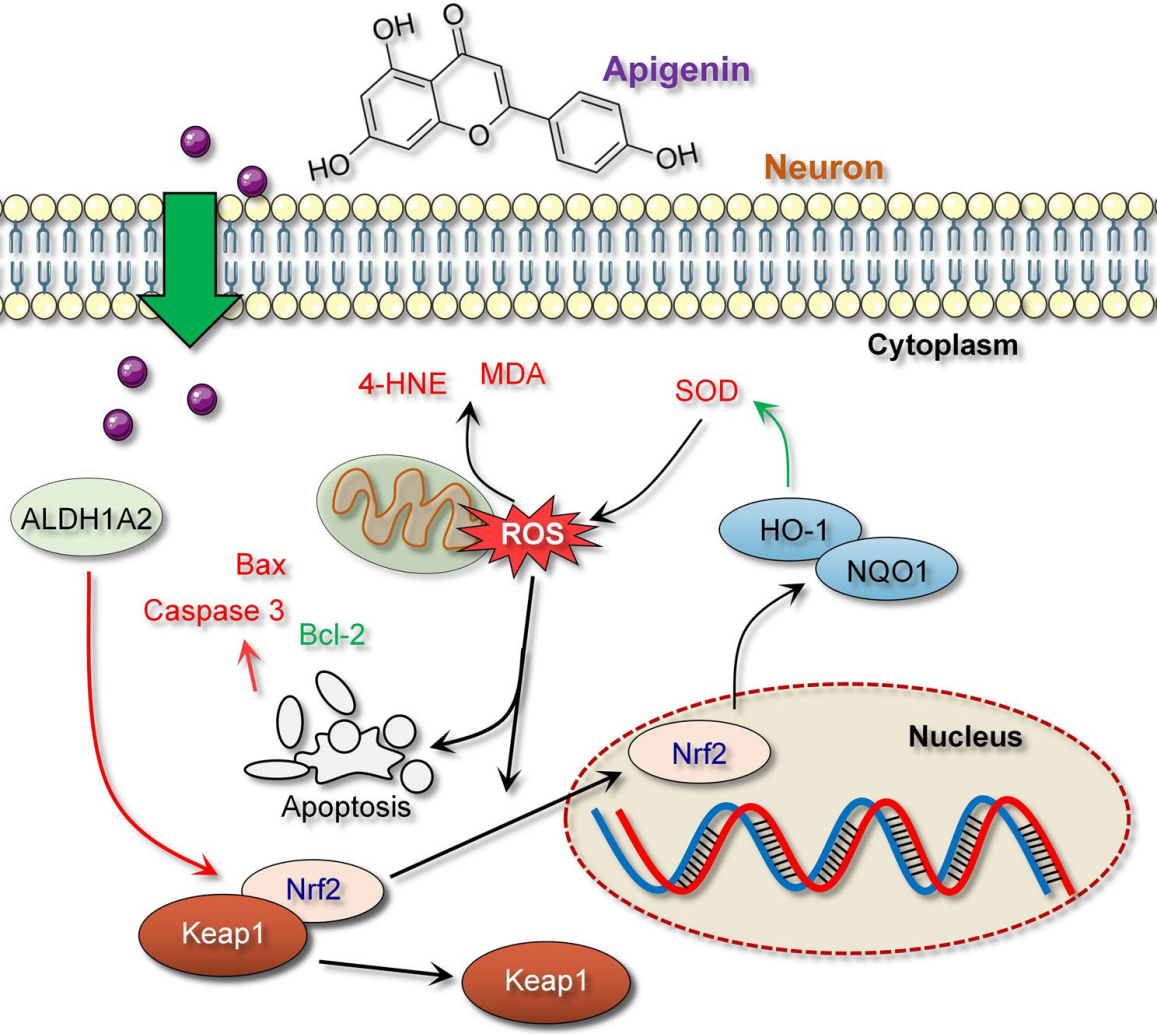

## Introduction

Amyotrophic lateral sclerosis (ALS) is a fatal neurodegenerative disease of the central nervous system characterized by the gradual degeneration and death of upper and lower motor neurons, leading to progressive muscle wasting and loss of strength (Feldman et al. 2022). Motor neuron damage leads directly to a decline in motor function, and patients in advanced stages often succumb to respiratory failure (Goutman et al. 2022). Therefore, understanding the pathogenesis of ALS and exploring viable therapeutic drugs or targets is critical.

The pathological hallmarks of ALS include neuroinflammation, increased oxidative stress (OS), and accelerated apoptotic processes (Soares et al. 2023). An important pathological feature in ALS is the aberration of TAR DNA-binding protein 43 (TDP-43), which is primarily located in the nucleus and is involved in RNA processing and gene regulation. However, numerous studies have confirmed that in ALS, TDP-43 translocates from the nucleus to the cytoplasm and forms protein aggregates that not only impair neuronal function but may also induce cell death (Oiwa et al. 2023). In addition, ALS pathology involves mutations in superoxide dismutase 1 (SOD1), the first gene associated with familial ALS. Mutations in SOD1 lead to enzyme inactivation or dysfunction, resulting in unstable aggregates that accumulate within cells, further exacerbating ALS and neuronal damage (Xu et al. 2022). Previous research has shown that the Tg(SOD1*G93A)1Gur/J mouse model, which harbors the G93A mutation of the SOD1 protein (Wintz et al. 2023), is an important tool for studying ALS because it exhibits neuropathological changes similar to those seen in human ALS and is widely used to understand the mechanisms by which SOD1 mutations contribute to increased OS and apoptosis (Mazala et al. 2024).

The neuronal antioxidant defense system consists mainly of enzymes such as SOD and glutathione (GSH), which work together to scavenge excessive reactive oxygen species (ROS), thereby protecting cells from oxidative damage (Dang et al. 2022). OS, characterized by the accumulation of ROS and other free radicals, leads to impaired expression and activity of antioxidant enzymes in ALS, rendering cells unable to efficiently scavenge ROS. This insufficiency increases the risk of lipid peroxidation and DNA damage (Djordjevic et al. 2023). Lipid peroxidation products such as malondialdehyde (MDA) and 4-hydroxynonenal (4-HNE) are biomarkers of OS, and elevated levels are closely associated with neuronal damage and death in the progression of ALS (Zhang et al. 2023). In addition, the accumulation of ROS can directly trigger apoptotic signaling pathways within cells (Lan et al. 2023), such as damage to mitochondrial membrane integrity, release of cytochrome c, and activation of the caspase cascade, which accelerates the pathological progression of ALS (Sharma et al. 2016). Previous studies have shown that aberrantly activated apoptotic pathways lead to excessive neuronal death involving dysregulation of the Bcl-2 family of proteins, with Bcl-2 acting as an anti-apoptotic protein while Bax and caspase-3 promote cell death (Yan et al. 2022).

Extensive research confirms that the Nrf2/ARE signaling pathway plays a critical role in the pathogenesis of many neurodegenerative diseases and serves as an important line of defense against OS (Li et al. 2022; Thiruvengadam et al. 2021). Nuclear factor erythroid 2-related factor 2 (Nrf2) is released from Keap1 under OS conditions and translocates to the nucleus to activate antioxidant response element (ARE)-driven gene expression (Ulasov et al. 2022). Recent studies by Davies et al. indicate that inactivation of Nrf2/ARE is key to the attenuation of antioxidant defenses and resultant oxidative damage to neuronal cells (Davies et al. 2021). Increasing the activity of the Nrf2 pathway may improve neurological function and prolong survival in ALS, suggesting that activation of this pathway may be a potential approach for the treatment of ALS. In addition, Nrf2 may also attenuate the aggregation of pathological proteins such as TDP-43 and SOD1 (Wang et al. 2022).

Apigenin (APG) is a naturally occurring flavonoid compound known for its antioxidant, anti-inflammatory, and antitumor properties (Waheed et al. 2023). Recent advances have highlighted the potential of apigenin in neuroprotective research, demonstrating its effects through various mechanisms, including activation of the Nrf2/ARE signaling pathway, thereby enhancing the cellular antioxidant defense system (Gaur et al. 2024; Thiruvengadam et al. 2021). Previous studies have confirmed that APG can protect against neuronal cell apoptosis in neurotoxin-induced rat models (Yadav et al. 2022). Aldehyde dehydrogenase 1 family member A2 (ALDH1A2) is an enzyme within the aldehyde dehydrogenase family that is responsible for oxidizing aldehydes to their corresponding carboxylic acids, thereby participating in metabolism and detoxification processes in the body (Zhang et al. 2021). Studies suggest that ALDH1A2 helps reduce oxidative damage caused by aldehydes that may be metabolically produced or introduced by external environmental stresses, making it a potential target for alleviating the pathological progression of ALS (Liang et al. 2017). Although the direct mechanisms by which APG modulates ALDH1A2 expression in ALS have not yet been fully elucidated, targeting ALDH1A2 with APG could provide a valuable therapeutic approach in counteracting ALS-related oxidative stress and cell apoptosis, supporting this study’s hypothesis. Thus, our focus on APG in this research arises from its proven ability to activate antioxidant defenses and its specific interactions with pathways relevant to ALS pathology, potentially offering therapeutic benefits in managing disease progression.

This study uses the Tg(SOD1*G93A)1Gur/J transgenic mouse model to understand the pathogenesis of ALS and explores the regulatory mechanisms of APG on ALDH1A2, thus laying the foundation for potential clinical applications of APG for ALS patients. This may also promote broader research and development of treatments for various neurodegenerative diseases.

### Materials and methods

### Experimental animals

In this study, B6SJL-Tg(SOD1*G93A)1Gur/J transgenic mice, which are widely used in ALS research, were used as an animal model of ALS. All mice were obtained from Nanjing Biomedical Research Institute of Nanjing University (Nanjing, China) and were acclimated for 1 week upon arrival with ad libitum access to food and water. Animals were housed under controlled conditions at 22 ± 2 °C with 55 ± 10% relative humidity and subjected to a 12-h light/12-h dark cycle. ALS mice were randomly divided into groups (*n* = 6): an untreated ALS model group and treatment groups receiving APG (purchased from Sigma-Aldrich (MO, USA) at 40 mg/kg and 80 mg/kg); wild-type (WT) mice (*n* = 6) received no treatment or intervention; the sh-ALDH1A2-treated group received bilateral intraperitoneal injections of 100µL lentivirus at a titer of 2 × 10^9^. Treatment groups received APG orally once daily from the beginning of the experiment until its completion, sh-ALDH1A2 treatment was initiated at symptom onset (day 90). Mouse weight and ALS Therapy Development Institute (ALSTDI) scores were monitored throughout the experiment. At the end of the experiment, mice were euthanized by intravenous injection of 100 mg/kg sodium pentobarbital, and spinal cord samples were collected under aseptic conditions. This study was approved by the Animal Ethics Committee of the First Affiliated Hospital of Nanchang University, approval number CDYFY-IACUC-202302QR086.

### Weight measurement and ALSTDI scoring

Weight monitoring began on day 90 after symptom onset and was performed every three days until day 120, the end of the experiment. ALSTDI scoring was used to assess motor function and disease progression in ALS model mice using a 0–4 scale from the ALS Therapy Development Institute, which quantifies motor function deterioration in the ALS mouse model as follows: 0 points: both hind limbs fully extended and maintained for more than 2 s during tail suspension; 1 point: incomplete extension of hind limbs with tremors observed when limbs were extended; 2 points: toe curling at least twice or dragging of hind limbs observed while climbing a 30 cm course; 3 points: clear paralysis or immobility of joints in both hind limbs, with forward movement entirely dependent on the forelimbs; 4 points: inability to right itself within 30 s, indicating a near-death state.

### Immunohistochemistry (IHC) analysis

After anesthesia, mice were perfused with 4% paraformaldehyde solution (Solarbio, Beijing, China) for fixation. Spinal cord tissues were then removed, fixed for 24 h, dehydrated, cleared, and embedded in paraffin. Tissue sections were deparaffinized followed by microwave antigen retrieval. Sections were incubated in blocking solution containing goat serum (Solarbio). After overnight incubation at 4 °C with primary antibody against TDP-43 (1:50, ab109535, Abcam, Cambridge, UK), sections were incubated with secondary antibody goat anti-rabbit IgG H&L (HRP) (1:2000, ab205718, Abcam) and visualized with DAB (Bioss, Beijing, China) under an inverted microscope (Leica Biosystems, Solms, Germany).

### Immunofluorescence (IF) analysis

Sections were incubated overnight at 4 °C with primary antibodies against NeuN (1:100, ab177487, Abcam) and ALDH1A2 (1:100, PA5-22377, Invitrogen, Thermo Fisher, MA, USA). Fluorescent secondary antibodies Goat anti-rabbit IgG H&L (Alexa Fluor^®^ 647) (1:200, ab150079, Abcam) for NeuN and Alexa Fluor^®^ 488 (1:200, ab150077, Abcam) for ALDH1A2 were used, and fluorescence signals were observed under a fluorescence microscope (Leica Biosystems).

### Cell culture and treatment

293FT and NSC34 cell lines from Procell (Wuhan, China) were cultured in DMEM containing 10% fetal bovine serum (FBS) at 37 °C. Transfection of a third-generation lentiviral packaging system (Sangon, Shanghai, China) containing pMDLg/pRRE (6 µg), pRSV-Rev (3 µg), pMD2.G (2 µg), and target plasmid sh-NC or sh-ALDH1A2 (PLVX-shRNA2-Puro, Sangon) into 293FT cells was performed using Lipofectamine 3000 (Invitrogen). After 6 h, the medium was replaced with DMEM containing 10% FBS. Supernatants were collected at 48 and 72 h post-transfection and concentrated by ultracentrifugation at 35,000 rpm for 100 min at 4 °C to a titer of 2 × 10^9^. NSC34 cells were infected with the lentivirus to establish stable cell lines. The SOD1*G93A vector was transfected into sh-ALDH1A2 NSC34 stable cells using Lipofectamine 3000 and the medium was changed after 4 h. For the APG-treated groups, NSC34 cells were pretreated 4 h prior to transfection with SOD1*G93A.

### CCK8 analysis

NSC34 cells were seeded at a density of 5 × 10^3^ cells/well in a 96-well plate and cultured at 37 °C for 24 h to allow adherence. 10 µL CCK-8 solution (Beyotime, Shanghai, China) was added to each well, and the cells were incubated at 37 °C for 4 h. Absorbance was measured at 450 nm using a microplate reader (Thermo Fisher).

### Apoptosis analysis

After harvesting, cells were washed three times with PBS and treated with 10 µL annexin V-FITC and 5 µL propidium iodide (PI) according to the manufacturer’s instructions for the annexin V-FITC/PI apoptosis kit. The procedure was performed in a dark, humid environment. Data were collected and analyzed using a flow cytometer (LSR-Fortessa, BD Biosciences, CA, USA).

### ROS analysis

ROS levels in spinal cord tissues and NSC34 cells from ALS model mice were detected using the fluorescent probe DCFDA (2’,7’-dichlorofluorescein diacetate, Thermo Fisher). Spinal cord tissues were minced in ice-cold PBS and digested with collagenase and DNAse for 30 min, followed by 10% FBS DMEM. The filtered suspension was centrifuged at 500 × g for 5 min. The resulting cell suspension and harvested NSC34 cells were incubated with 10 µM DCFDA at 37 °C for 30 min to allow the probe to react with intracellular ROS and generate fluorescence. DCF fluorescence intensity was measured using a flow cytometer (BD Biosciences).

### ELISA analysis

Collected mouse spinal cord tissues were crushed in liquid nitrogen and homogenized in RIPA lysis buffer. The lysate and supernatants of NSC34 cells were centrifuged at 500×g, and the activities of SOD and ALDH and the concentrations of MDA and 4-HNE were measured using an ELISA kit (Solarbio) according to the manufacturer’s instructions. Absorbance was read at specific wavelengths using a microplate reader to quantify the concentrations of target proteins in tissue and cell samples.

### qRT-PCR analysis

Total RNA was extracted from mouse spinal cord tissues or NSC34 cell lines using TRIzol (Invitrogen) according to the manufacturer’s instructions. cDNA was synthesized using 2×NovoScript^®^ Plus 1st Strand cDNA Synthesis SuperMix (Novoprotein, Suzhou, China). qRT-PCR reactions were performed using NovoStart^®^ SYBR qPCR SuperMix Plus (Novoprotein). PCR conditions were as follows: 95 °C for 1 min, followed by 40 cycles of 95 °C for 20 s, 56 °C for 20 s, and extension for 38 s. Data analysis was performed using the 2^−ΔΔCT^ method, normalized to the housekeeping gene GAPDH. Primers used for qRT-PCR are listed in Table 1.

**Table 1:** Primer sequence of qRT-PCR

Gene	Forward primers (5′-3′)	Reverse primers (5′-3′)
GAPDH	TGGAAAGCTGTGGCGTGATG	TACTTGGCAGGTTTCTCCAGG
ALDH1A2	ATCGCTTCTCACATCGGCAT	ACAGCGTAGTCCAAGTCAGC
Nrf2	CCAGCACATCCAGACAGACAC	GATATCCAGGGCAAGCGACTC
HO-1	CAGGGTGACAGAAGAGGCTAAGAC	TTGTGTTCCTCTGTCAGCATCAC
NQO1	AGGATGGGAGGTACTCGAATC	AGGCGTCCTTCCTTATATGCTA

Western blot analysis

Total protein was extracted from treated mouse spinal cord tissues and NSC34 cells using RIPA lysis buffer (Beyotime). Protein concentrations were determined using a BCA protein assay kit (Beyotime). Denatured proteins were loaded onto SDS-PAGE gels for electrophoretic separation and then transferred to PVDF membranes. After transfer, the membranes were blocked with 5% nonfat milk in TBST (Solarbio) for 1 h and then incubated overnight at 4 °C with primary antibodies against ALDH1A2 (1:500), Bcl-2 (1: 2000, ab182858, Abcam), caspase 3 (1:5000, ab32351, Abcam), Bax (1:1000, ab32503, Abcam), Nrf2 (1:500, PA5-27882, Invitrogen), HO-1 (1:1000, PA5-77833, Invitrogen), and NQO1 (1:3000, ab80588, Abcam). After three washes with TBST, the membranes were incubated with HRP-conjugated secondary antibodies (1:2000, ab205718, Abcam) for 1 h. Protein bands were visualized using an ECL detection system (Thermo Fisher) and quantitated using Image J software (National Institutes of Health [NIH], MD, USA).

### Data analysis

Data were presented as mean ± standard deviation (Mean ± SD) and analyzed using GraphPad Prism 8.0. Comparisons between multiple groups were performed using one-way ANOVA with Bonferroni’s post-hoc test. A p-value < 0.05 was considered statistically significant.

## Results

### APG treatment slows weight decline in ALS mice

**Fig. 1:**
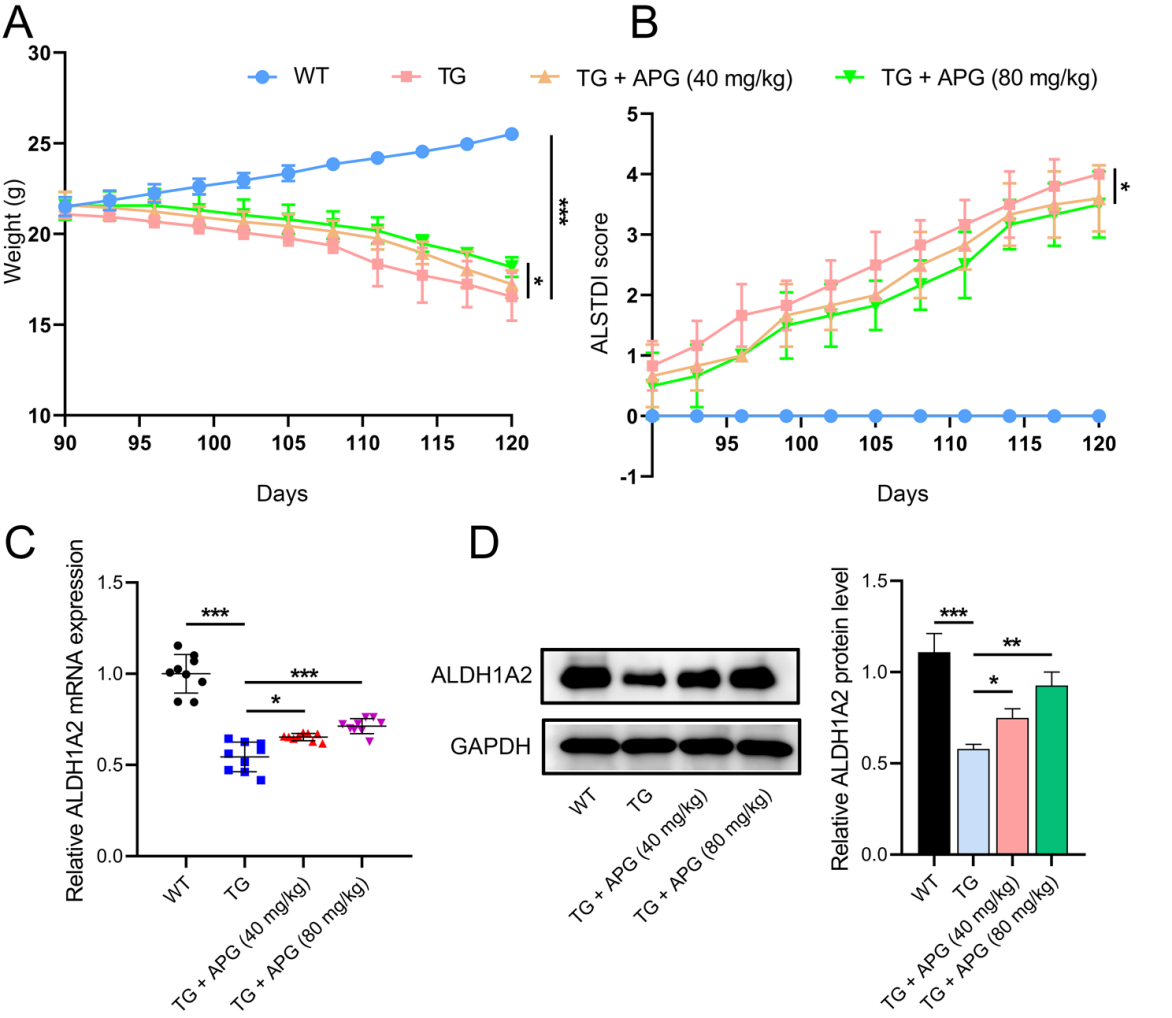
Effects of APG on body weight and ALDH1A2 expression in ALS mice. **A** Measurement of body weight in APG-treated ALS mice. **B** Assessment of behavioral effects using the ALSTDI scoring system in APG-treated ALS mice. **C** qRT-PCR analysis of the effect of APG on ALDH1A2 mRNA levels in spinal cord tissues of ALS mice. **D** Western blot analysis showing the influence of APG on ALDH1A2 protein levels in spinal cord tissues of ALS mice. *P < 0.05, **P < 0.01, *P < 0.001

Using Tg(SOD1*G93A)1Gur/J transgenic mice as an ALS model, we investigated the therapeutic potential of APG. Throughout the study, wild-type (WT) mice showed consistent weight gain, whereas the ALS model group and the APG-treated group both experienced weight loss, with the latter showing a slower rate of decline (Fig. 1A). ALSTDI scores showed that APG-treated mice consistently scored lower than untreated ALS mice, suggesting a mitigation of motor function decline (Fig. 1B). After treatment, ALS mice showed a significant reduction in ALDH1A2 mRNA and protein levels in spinal cord tissues, while an upregulation of ALDH1A2 was observed in the APG-treated groups, especially at a dose of 80 mg/kg (Fig. 1C, D), highlighting a potential key role of ALDH1A2 in the protective effects of APG against ALS.

### APG reduces OS and apoptosis in ALS mice

**Fig. 2:**
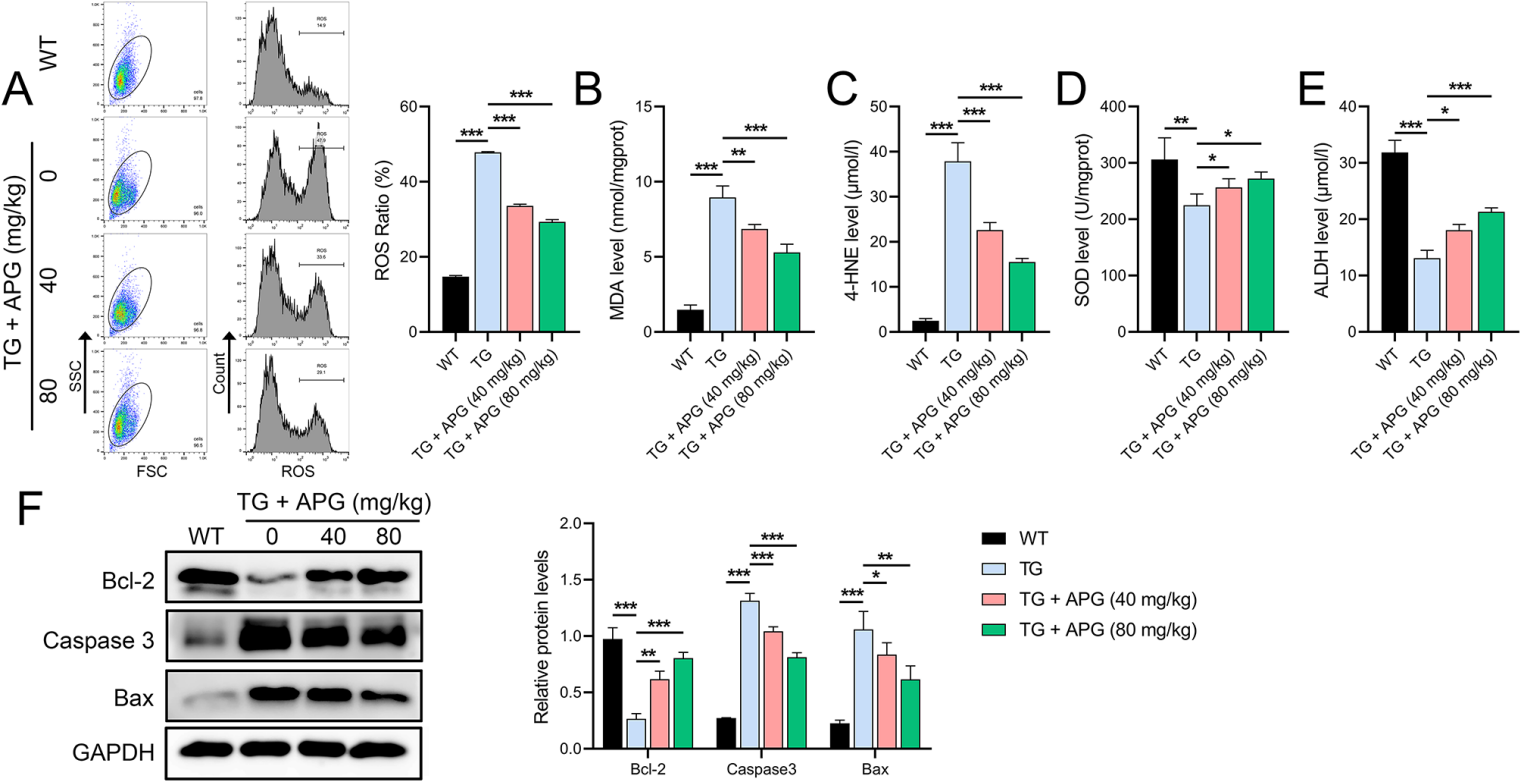
OS and apoptosis markers in spinal cord of ALS mice. **A** Flow cytometry analysis of ROS levels in spinal cord tissues of APG-treated ALS mice. **B**-**E** ELISA to quantify the levels of MDA, 4-HNE, SOD and ALDH in the spinal cord tissues of ALS mice under APG treatment. **F** Western blot analysis of the levels of apoptosis-related proteins (Bcl-2, caspase-3, Bax) in the spinal cords of APG-treated ALS mice. *P < 0.05, **P < 0.01, *P < 0.001

Further investigation of the effects of APG revealed its impact on OS and apoptosis. Increased levels of ROS and aldehyde markers MDA and 4-HNE were observed in spinal cord homogenates from ALS mice (Fig. 2A-C), along with decreased levels of SOD and ALDH (Fig. 2D, E). APG treatment significantly ameliorated these changes. In addition, decreased Bcl-2 and increased Bax and caspase-3 levels in ALS mice indicated increased apoptosis, which was significantly improved by APG treatment, especially at the higher dose of 80 mg/kg (Fig. 2F).

### APG ameliorates ALS-related protein expression anomalies

**Fig. 3:**
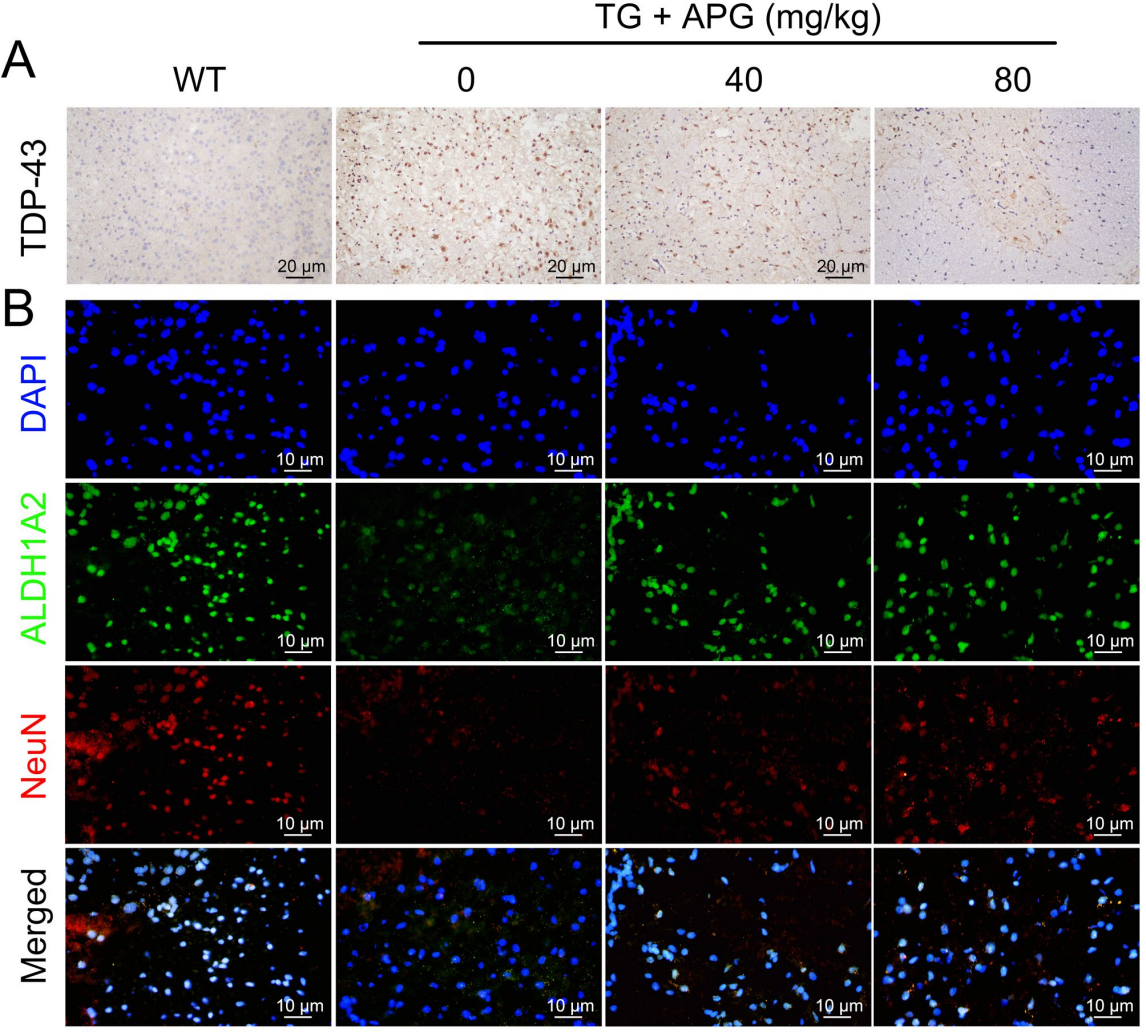
Mitigation of ALS pathology in the lumbar spinal cord anterior horn of ALS mice by APG. **A** IHC analysis of the effect of APG on the expression of the ALS-characteristic protein TDP-43 in the ventral lumbar spinal cord anterior horn of ALS mice. **B** IF analysis of the distribution of NeuN and ALDH1A2 in the ventral lumbar spinal cord anterior horn tissue of APG-treated ALS mice

IHC results showed high cytoplasmic expression of the ALS hallmark protein TDP-43 in the lumbar spinal cord anterior horn of ALS mice, specifically in ventral motor neurons, which was significantly reduced after APG treatment (Fig. 3A). IF assays revealed a marked reduction in NeuN and ALDH1A2 distribution in the lumbar spinal cord anterior horn cells of ALS mice, which was significantly alleviated by APG treatment, especially at the higher dose of 80 mg/kg (Fig. 3B). These results suggest a critical role for ALDH1A2 in mitigating neuronal damage in APG-treated ALS mice.

### Dynamic expression of ALDH1A2 across ALS progression stages

**Fig. 4:**
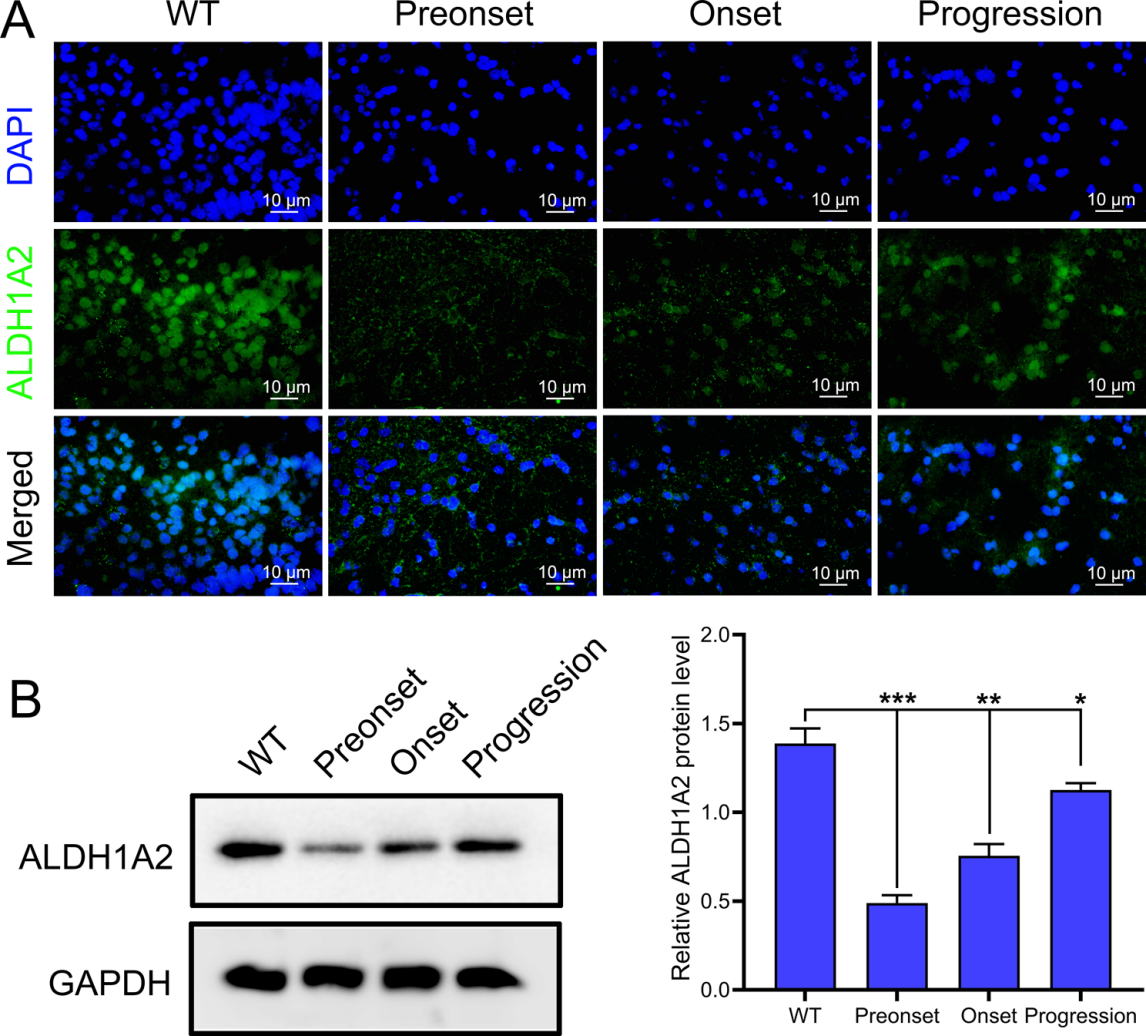
ALDH1A2 expression at different stages of ALS in mice. **A** IF analysis showing the expression of ALDH1A2 at the preonset, onset, and progression stages of ALS. **B** Western blot analysis of ALDH1A2 protein levels at different stages of the disease. *P < 0.05, **P < 0.01, *P < 0.001

Using the Tg(SOD1*G93A)1Gur/J mouse model, we investigated the potential role of ALDH1A2. Compared with WT mice, ALDH1A2 expression was significantly reduced in the preonset group of Tg(SOD1*G93A)1Gur/J mice; it increased at the onset of ALS and further increased during the progression stages of the disease (Fig. 4). These results suggest that ALDH1A2 may be involved in the pathogenesis of ALS and is closely related to the cellular OS response mechanism.

### APG enhances viability and reduces apoptosis in NSC34 cells

**Fig. 5:**
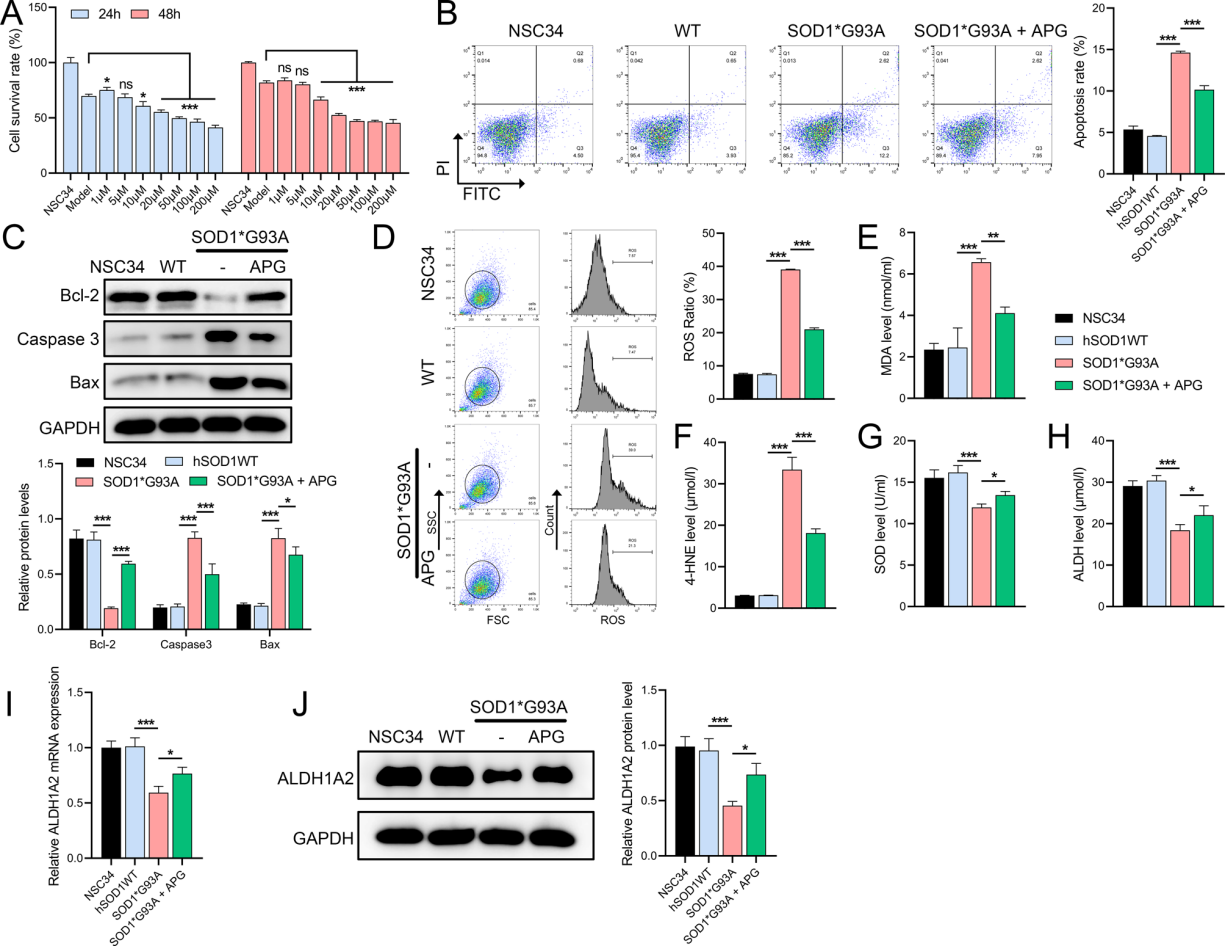
Effect of APG on viability and apoptosis in NSC34 neuronal cells. **A** CCK8 assay to evaluate the effects of different doses of APG on the viability of NSC34 cells in vitro. **B** Flow cytometry analysis of apoptosis in NSC34 cells harboring the SOD1*G93A mutation treated with APG. **C** Western blot analysis of apoptosis-related proteins (Bcl-2, caspase-3, Bax) in SOD1*G93A mutant NSC34 cells treated with APG. **D** Flow cytometry assessment of ROS levels in SOD1*G93A mutant NSC34 cells under APG treatment. **E**-**H** ELISA for quantification of OS markers (MDA, 4-HNE, SOD, ALDH) in SOD1*G93A mutant NSC34 cells treated with APG. **I** qRT-PCR analysis of ALDH1A2 mRNA levels, and (J) Western blot analysis of ALDH1A2 protein levels in APG-treated SOD1*G93A mutant NSC34 cells. *P < 0.05, **P < 0.01, *P < 0.001

In vitro studies using cultured NSC34 neuronal cells transfected with the SOD1*G93A mutation showed that higher doses of APG (10µM) significantly reduced cell viability at 24 and 48 h (Fig. 5A). A safe dose of 1µM APG was selected for 24-h treatment, which effectively improved cell viability. Flow cytometry showed that the SOD1*G93A mutation resulted in extensive apoptosis in NSC34 cells, which was significantly reduced by APG treatment (Fig. 5B). Western blot analysis further showed that APG partially counteracted the mutation-induced decrease in Bcl-2 protein levels and increase in Bax and caspase-3 levels (Fig. 5C). In addition, flow cytometry showed that APG reduced ROS accumulation caused by the SOD1*G93A mutation (Fig. 5D). ELISA results showed that the mutation increased the levels of aldehyde markers MDA and 4-HNE and decreased the levels of SOD and ALDH, but these were significantly attenuated by APG treatment (Fig. 5E-H). Furthermore, the mutation-induced reductions in ALDH1A2 mRNA and protein levels were significantly reduced by APG (Fig. 5I, J), confirming the hypothesis from in vivo experiments that ALDH1A2 plays a role in the protective mechanisms of APG in ALS models.

### Impact of ALDH1A2 knockdown and APG treatment on ALS model

**Fig. 6:**
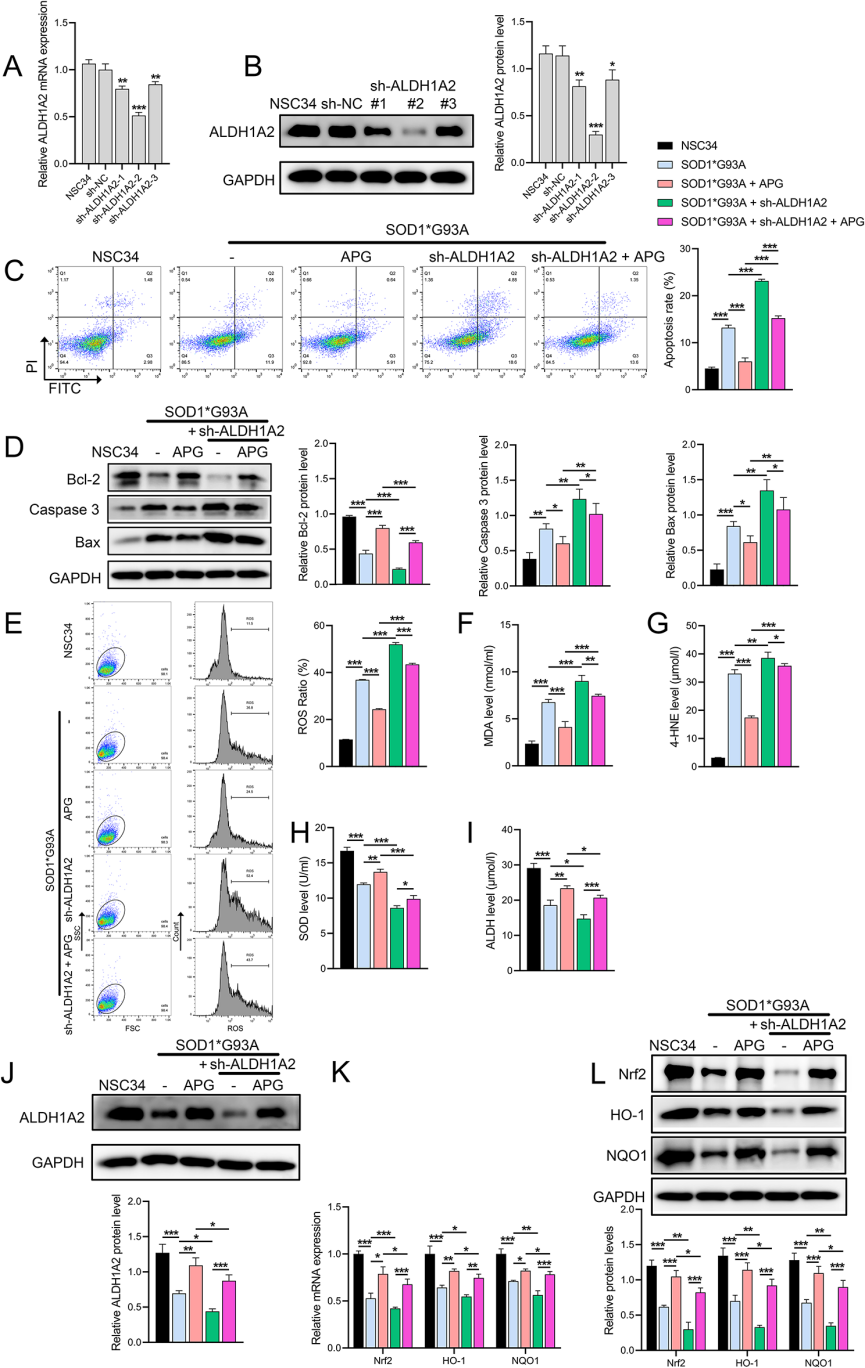
Effects of ALDH1A2 knockdown and APG treatment on ALS model and OS markers. **A**-**B** Validation and selection of the most effective sh-ALDH1A2 to suppress ALDH1A2 expression by qRT-PCR and Western blot analysis. **C** Flow cytometry to evaluate apoptosis in NSC34 cells treated with APG and sh-ALDH1A2. **D** Western blot to evaluate the levels of apoptosis-related proteins (Bcl-2, caspase-3, Bax) in NSC34 cells treated with both APG and sh-ALDH1A2. **E** Flow cytometric analysis of ROS levels in treated NSC34 cells. **F**-**I** ELISA for quantification of OS markers (MDA, 4-HNE, SOD, ALDH) in NSC34 cells treated with APG and sh-ALDH1A2. **J** Western blot analysis of ALDH1A2 protein levels. **K**-**L** qRT-PCR and Western blot analysis of Nrf2, HO-1, NQO1 mRNA and protein levels in NSC34 cells treated with APG and sh-ALDH1A2. *P < 0.05, **P < 0.01, *P < 0.001

We constructed three sh-ALDH1A2 lentiviruses and infected NSC34 cells. RT-qPCR and Western blot results showed that all three effectively suppressed ALDH1A2 expression, with ALDH1A2-sh2 showing the most significant inhibition (Fig. 6A, B) and used for further experiments. The results showed that sh-ALDH1A2 exacerbated apoptosis of NSC34 cells induced by the SOD1*G93A mutation, decreased Bcl-2 protein levels, and increased Bax and caspase-3 levels, whereas APG treatment effectively attenuated these effects (Fig. 6C, D). Furthermore, sh-ALDH1A2 exacerbated the increase in ROS and aldehyde markers MDA and 4-HNE and the decrease in SOD and ALDH caused by the SOD1*G93A mutation, but APG alleviated these effects (Fig. 6E-I). In the process, APG reversed the suppression of ALDH1A2 mRNA and protein levels caused by sh-ALDH1A2 (Fig. 6J, K), confirming that ALDH1A2 is a key gene in the protective mechanism of APG. We also analyzed the key OS signals Nrf2/ARE; the results showed that the mRNA and protein levels of Nrf2, HO-1, and NQO1 were significantly decreased in the SOD1*G93A mutation, but increased under APG treatment. Sh-ALDH1A2 intervention further suppressed these protein expressions, but APG partially reversed this suppression (Fig. 6L, M).

### Final verification of APG and sh-ALDH1A2 combination in ALS mouse model

**Fig. 7:**
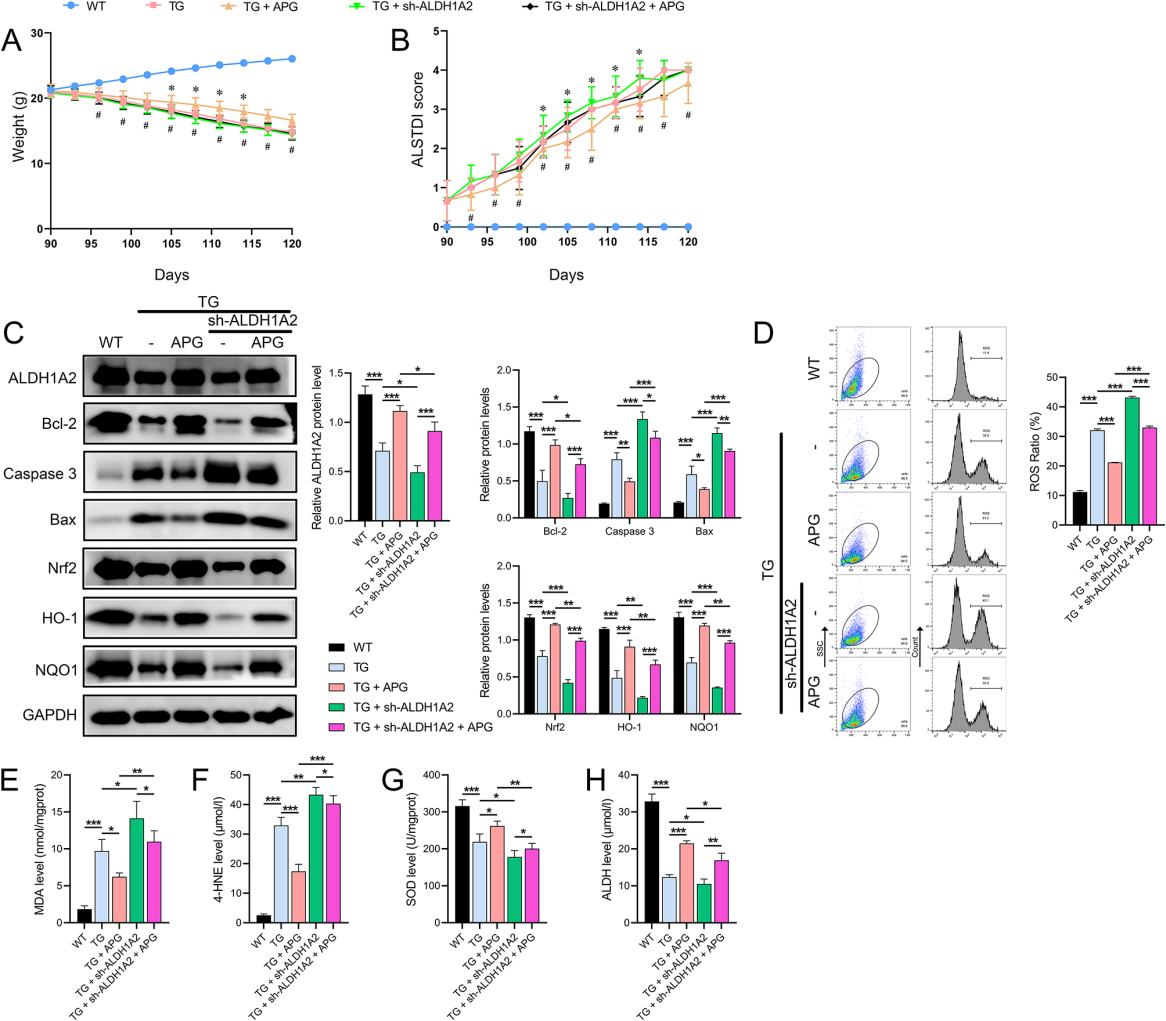
Combined effects of APG and sh-ALDH1A2 on in vivo ALS mouse model. **A** Body weight measurements in ALS mice treated with APG and sh-ALDH1A2. *, TG v.s TG + sh-ALDH1A2 group; #, TG + APG v.s TG + sh-ALDH1A2 + APG group. **B** Behavioral effects assessed by ALSTDI scores in ALS mice treated with APG and sh-ALDH1A2. **C** Western blot analysis of protein levels (ALDH1A2, Bcl-2, Caspase-3, Bax, Nrf2, HO-1, NQO1) in spinal cord tissues of treated ALS mice. **D** Flow cytometric analysis of ROS levels. **E**-**H** ELISA quantifying OS markers (MDA, 4-HNE, SOD, ALDH) in spinal cord tissues of treated ALS mice. #*P* < 0.05, *P < 0.05, **P < 0.01, *P < 0.001

Finally, the combined use of APG and sh-ALDH1A2 in an ALS mouse model further validated the in vivo mechanism of action of APG. Results showed that during APG treatment, the body weight of ALS mice improved and their ALSTDI scores decreased. In contrast, ALS mice treated with sh-ALDH1A2 had lower body weights between days 105 and 114 and higher ALSTDI scores between days 102 and 114 compared to ALS mice. Compared to the APG-treated group, the sh-ALDH1A2-treated group showed continuous weight loss from day 96 and increasing ALSTDI scores from day 93 (Fig. 7A, B). Moreover, sh-ALDH1A2 further enhanced the reduction of ALDH1A2 and Bcl-2 protein levels and the increase of Bax and caspase 3 levels in the spinal cord tissues of Tg(SOD1*G93A)1Gur/J mice, and APG effectively alleviated these changes (Fig. 7C). In addition, APG not only alleviated the increased levels of ROS and aldehyde markers MDA and 4-HNE and the decreased levels of SOD and ALDH in spinal cord tissues of ALS mice, but also substantially offset the exacerbating effects of sh-ALDH1A2 on these OS markers (Fig. 7D-H).

**Fig. 8:**
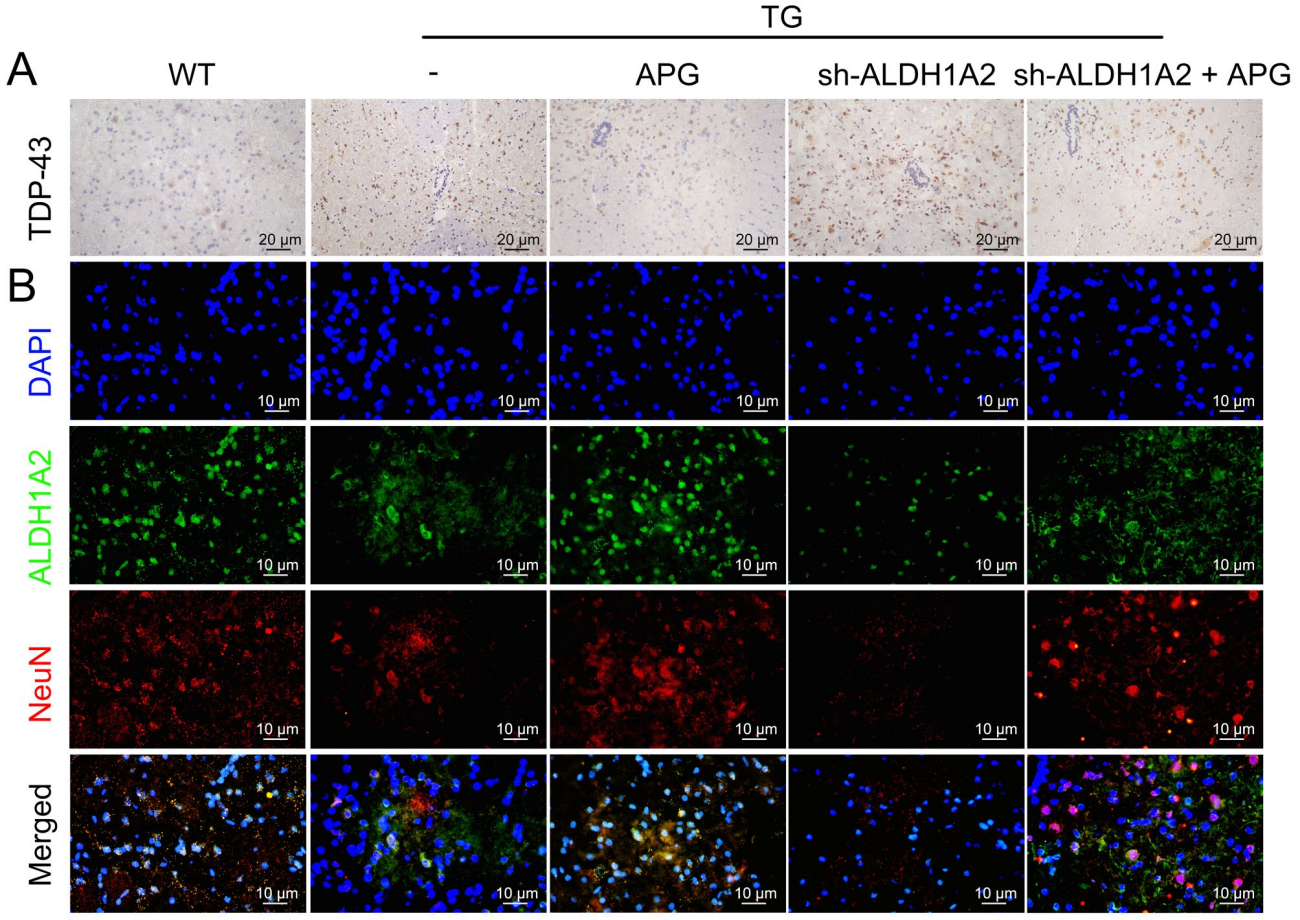
IHC and IF responses to APG and sh-ALDH1A2 in the spinal cord of ALS mice. **A** IHC analysis evaluating the expression of TDP-43 in the spinal cord tissues of ALS mice treated with both APG and sh-ALDH1A2. **B** IF analysis evaluating the distribution of NeuN and ALDH1A2 in the spinal cord tissues of ALS mice treated with APG and sh-ALDH1A2

In addition, immunohistochemical results showed that APG treatment not only alleviated TDP-43 in ALS mouse tissues, but also significantly counteracted the exacerbating effects of sh-ALDH1A2 on ALS mice (Fig. 8A). Furthermore, IF results showed that APG treatment not only ameliorated the reduced distribution of NeuN and ALDH1A2 in ALS mouse tissues, but also significantly counteracted the exacerbating effects of sh-ALDH1A2 on ALS (Fig. 8B).

## Discussion

ALS is a progressive neurodegenerative disease that affects neurons in the brain and spinal cord, resulting in the loss of muscle control (Akcimen et al. 2023). In this study, we have demonstrated for the first time the significant protective effects of APG in delaying the progression of ALS using the Tg(SOD1*G93A)1Gur/J mouse model, highlighting its pronounced mitigating effect on disease progression. This finding, compared to other ALS therapeutic interventions reported in the literature, suggests that APG has significant therapeutic potential (Ilieva et al. 2023; Soares et al. 2023). Under the influence of APG, the rate of weight loss in ALS mice was slowed, possibly reflecting the potential effects of APG in preserving muscle function and mass. Progressive weight loss is a common and severe clinical manifestation in ALS patients, often associated with rapid disease progression and poor prognosis (Shefner et al. 2022). Concurrently, results from the ALSTDI scoring also demonstrated the symptomatic relief provided by APG, suggesting that its effects could be significant in improving the quality of life and overall survival of ALS patients.

ALDH1A2, a key antioxidant enzyme, has been widely reported to play a protective role in several neurodegenerative diseases (Kataoka et al. 2023; Khatib et al. 2020). Previous research has established ALDH1A2 as a therapeutic target for ALS (Zhu et al. 2020). Our molecular analyses further revealed that APG treatment significantly upregulated the mRNA and protein expression and distribution of ALDH1A2 in the spinal cord tissues of ALS mice. Thus, the upregulation of ALDH1A2 suggests that APG may exert its protective effects by enhancing antioxidant defenses in mice. The positive regulation of ALDH1A2 by APG may be a key mechanism by which it moderates the pathological progression of ALS. In addition, aldehyde accumulation has been reported to be an important cause of OS; ALDH enzymes specifically metabolize and degrade aldehyde compounds to their corresponding acids, a process that helps reduce intracellular toxicity and OS (Park et al. 2023). Therefore, we analyzed OS levels and aldehyde content in ALS mouse spinal cord tissues and observed that APG, especially at higher doses (80 mg/kg), at least partially restored high levels of OS (increased ROS, decreased SOD and ALDH) and aldehyde accumulation (increased MDA and 4-HNE) to normal levels, not only demonstrating APG’s role as an antioxidant in various diseases, but also establishing the central role of ALDH1A2 in APG’s alleviation of ALS symptoms. Thus, our study monitored changes in ALDH1A2 expression during disease progression and showed that ALDH1A2 expression was downregulated in pre-symptomatic ALS mice, possibly due to an inadequate initial cellular response to the disease. As an antioxidant enzyme, its upregulation in the early stages of the disease may reflect cellular attempts to restore OS balance. This upregulation could be transient or insufficient to combat ongoing neural damage, but it indicates the potential protective role of ALDH1A2 in the pathogenesis of ALS. In later stages of the disease, possibly due to cellular adaptation to prolonged OS, ALDH1A2 expression was found to increase. This increase might be a compensatory mechanism intended to bolster cellular antioxidant defenses.

Recent studies have confirmed that apoptosis plays a key role in the onset and progression of ALS, and OS is closely related to apoptotic processes (Calderaro et al. 2022), as shown by our results, where APG alleviated OS in ALS and NSC neuronal cells while inhibiting cell apoptosis and activation of apoptosis-related pathways. This could be due to excessive ROS triggering mitochondrial pathways leading to activation of apoptotic effectors such as caspase 3 and imbalance in Bcl-2/Bax ratio (Liang et al. 2023). The dual antioxidant and anti-apoptotic effects of APG reduce ROS accumulation and inhibit the activation of apoptotic pathways. In addition, in ALS pathological studies, abnormal aggregation of TDP-43 protein is a hallmark biomarker that plays a key role in disease progression (Lepine et al. 2022). In our study, we found that TDP-43 expression was significantly elevated in the spinal cord tissues of untreated ALS mice, consistent with the pathological changes observed in ALS patients. However, after APG treatment, the abnormal TDP-43 expression was significantly alleviated, suggesting that APG may exert its neuroprotective effects by regulating the metabolism or function of TDP-43. In addition, untreated ALS mice showed a significant reduction in the distribution of NeuN and ALDH1A2, which are associated with neuronal damage and death (Li et al. 2021). Notably, APG treatment significantly ameliorated this phenomenon, especially in the high-dose treatment group, where NeuN and ALDH1A2 levels were substantially restored. These observations highlight the potential role of APG in mitigating ALS-related neurodegenerative changes by preventing abnormal TDP-43 aggregation and restoring normal neuronal protein distribution, providing a potential therapeutic strategy to improve clinical symptoms and prolong survival in ALS patients.

In conclusion, to confirm the role of ALDH1A2 in the mechanism of action of APG, our reversal experiments observed that knockdown of ALDH1A2 expression exacerbated OS, aldehyde accumulation and apoptosis in ALS mice or in vitro NSC34 cells, and inhibited the Nrf2/ARE signaling pathway (decreased expression of Nrf2, NQO1), while APG treatment not only attenuated OS and apoptosis in both in vivo and in vitro models of ALS, but also reduced the promotive effects of ALDH1A2 knockdown on ALS progression, further confirming that ALDH1A2 is positively regulated by APG to attenuate ALS progression. Here, we first confirm that these effects of ALDH1A2 are achieved through the activation of key OS signals Nrf2/ARE, as Nrf2/ARE is a critical regulatory system for cellular defense against OS and maintenance of redox balance (Liu et al. 2022). Existing evidence shows that in neurodegenerative diseases, activation of Nrf2 can alleviate neuronal damage caused by OS and aldehyde accumulation, as Nrf2 binding to ARE induces the expression of various antioxidant genes such as HO-1 and NQO1, thereby enhancing the scavenging of ROS or other free radicals (Egbujor et al. 2023).

This study has limitations. First, we did not obtain clinical sample data on the efficacy of APG treatment. Second, existing evidence shows that the antioxidant enzyme ALDH1A2 regulates downstream genes or pathways far beyond Nrf2/ARE, which was not further explored in this article. Third, both the Nrf2/ARE pathway and OS are key pathways for ferroptosis, an important form of apoptosis, which was not explored in this article. Fourth, the effects of APG on other antioxidant defense mechanisms were also not fully investigated. Fifth, this study primarily focuses on the therapeutic effects of APG and the underlying mechanism involving ALDH1A2 during the early stages of ALS, without exploring the effects of APG treatment in the middle or late stages of the disease. Sixth, our experiments using spinal cord homogenates from ALS model mice included whole spinal cord tissue, meaning that cells other than motor neurons were also analyzed, which could influence interpretations specific to motor neuron pathology. Finally, this study mainly relied on the Tg(SOD1*G93A)1Gur/J transgenic mouse model to simulate the pathological process of ALS. Although this model is widely used in ALS research, it only covers some pathological features of ALS, especially familial ALS related to SOD1 mutations, and may not fully represent more common sporadic cases of ALS.

## Conclusion

Our study systematically investigated the therapeutic potential of APG in an ALS mouse model and the molecular mechanisms involved. The results suggest that APG can significantly slow the pathological progression of ALS, mainly by alleviating neuronal injury, inhibiting cell apoptosis, and improving OS responses. APG’s upregulation of ALDH1A2 played a key role in enhancing cellular antioxidant defense capabilities by activating the Nrf2/ARE signaling pathway Graphical abstract. In addition, APG significantly modulated the abnormal expression of TDP-43 and its associated neuronal damage, thereby ameliorating disease-related neuropathological changes. The multiple protective effects of APG demonstrate its comprehensive ability against the ALS disease process and highlight its potential to modulate key molecules and pathways. Further research into the therapeutic mechanisms of APG and its clinical application may provide new treatment strategies for ALS patients, offering hope for the management of this complex disease.

## Data Availability

The datasets used and/or analysed during the current study are available from the corresponding author on reasonable request.

## Abbreviations

ALS: Amyotrophic Lateral Sclerosis
APG: Apigenin
OS: Oxidative Stress
SOD1: Superoxide Dismutase 1
ALSTDI: ALS Therapy Development Institute
IHC: Immunohistochemistry
IF: Immunofluorescence
qRT: PCR-Quantitative Reverse Transcription Polymerase Chain Reaction
NSC34: A hybrid cell line derived from mouse neuroblastoma and motor neurons
shRNA: Short Hairpin RNA
WT: Wild Type
Bcl: 2-B-cell lymphoma 2
MDA: Malondialdehyde
4: HNE-4-Hydroxynonenal
Nrf2: Nuclear Factor Erythroid 2-Related Factor 2
ARE: Antioxidant Response Element
GSH: Glutathione
ROS: Reactive Oxygen Species
HO: 1-Heme Oxygenase-1
NQO1: NAD(P)H Quinone Dehydrogenase 1
Bax: Bcl-2-associated X protein
ALDH: Aldehyde Dehydrogenase
DCFDA: 2’,7’-Dichlorofluorescin Diacetate
PI: Propidium Iodide
ECL: Enhanced Chemiluminescence
DMEM: Dulbecco’s Modified Eagle Medium
FBS: Fetal Bovine Serum
PBS: Phosphate-buffered Saline
ELISA: Enzyme-Linked Immunosorbent Assay
HRP: Horseradish Peroxidase
TBST: Tris-buffered Saline with Tween 20
PVDF: Polyvinylidene Difluoride
RNA: Ribonucleic Acid
DNA: Deoxyribonucleic Acid
GAPD: Glyceraldehyde 3-phosphate Dehydrogenase
